# Affect and Arousal in Insomnia: Through a Lens of Neuroimaging Studies

**DOI:** 10.1007/s11920-020-01173-0

**Published:** 2020-07-13

**Authors:** Julian E. Schiel, Florian Holub, Roxana Petri, Jeanne Leerssen, Sandra Tamm, Masoud Tahmasian, Dieter Riemann, Kai Spiegelhalder

**Affiliations:** 1 Department of Psychiatry and Psychotherapy, Faculty of Medicine, Medical Center – University of Freiburg, Hauptstraße 6, 79104 Freiburg, Germany; 2 Department of Sleep and Cognition, Netherlands Institute for Neuroscience, Amsterdam, The Netherlands; 3 Department of Integrative Neurophysiology, Center for Neurogenomics and Cognitive Research (CNCR), Amsterdam Neuroscience, VU University Amsterdam, Amsterdam, The Netherlands; 4 Department of Clinical Neuroscience, Karolinska Institutet, Stockholm, Sweden; 5 Department of Psychiatry, Oxford University, Oxford, UK; 6 Institute of Medical Science and Technology, Shahid Beheshti University, Tehran, Iran

**Keywords:** Insomnia, Neuroimaging, Affect and arousal, Salience network, Default mode network, Limbic circuit

## Abstract

**Purpose of Review:**

Previous research has struggled with identifying clear-cut, objective counterparts to subjective distress in insomnia. Approaching this discrepancy with a focus on hyperarousal and dysfunctional affective processes, studies examining brain structures and neural networks involved in affect and arousal are reviewed and conclusions for an updated understanding of insomnia are drawn.

**Recent Findings:**

Recent studies found that amygdala reactivity, morphometry and adaptation in insomnia are altered, indicating that processing of negative stimuli is intensified and more lasting. Also, patients with insomnia show aberrant connectivity in the default mode network (DMN) and the salience network (SN), which is associated with subjective sleep disturbances, hyperarousal, maladaptive emotion regulation and disturbed integration of emotional states. The limbic circuit is assumed to play a crucial role in enhanced recall of negative experiences.

**Summary:**

There is reason to consider insomnia as a disorder of affect and arousal. Dysregulation of the limbic circuit might perpetuate impaired connectivity in the DMN and the SN. However, the interplay between the networks is yet to be researched.

## Introduction

Insomnia is a sleep disorder characterized by difficulties in initiating and maintaining sleep. Typically, these nighttime disturbances go along with various daytime impairments, such as mood swings, deficits in attention and memory problems [1]. With a point prevalence of 6–10% [2] and an incidence rate of 4% per year [3], insomnia is the most prevalent sleep disorder and one of the most widespread diseases in western industrialized countries. Moreover, demographic trends in these countries suggest that the societal relevance of insomnia might even increase in the future [4], given the fact that the prevalence rises with age [2, 5]. Insomnia is also closely linked to depression, as epidemiological (insomnia as a predictor of depression, see [6, 7]) and neurobiological (for a systematic review, see [8•]) research suggests. It seems therefore particularly important to understand underlying features of insomnia related to mood and affect.

## Insomnia: A Matter of Perspective?

As variously suggested by previous research, principal insomnia symptoms like impaired sleep continuity and daytime dysfunction mainly become tangible in subjective measures [1, 9, 10]. Exemplarily, polysomnography studies (for a PSG meta-analysis, see [10]) show only marginal differences in objective sleep duration between patients with insomnia and healthy good sleepers (HGS). Objective measures of cognitive performance [11] and neuroimaging studies [12] so far remain inconsistent, struggling with effect sizes that cannot be reliably detected with the available sample size. The absence of clear-cut objective, pathophysiological counterparts to subjective impairments makes understanding of insomnia way more challenging. One approach to overcome this apparent contradiction is rethinking objective measurement: Do we focus on the right parameters applying appropriate methods? Do we analyze data in a sufficiently differentiated way? In the recent years, multiple studies took this approach with promising results: For example, a more differentiated view on sleep parameters and polysomnography allowed detection of fragmented REM sleep in patients with insomnia [13, 14]; neuroimaging was expanded by examination of white matter integrity [15, 16]. At this, it must be remarked that systematic re-differentiating of methodical and analytical strategies might run the risk of contradicting the principle of falsification, especially if not backed by strong theoretical considerations [17]. This remark also applies for subtyping approaches aiming for re-differentiation of the concept of insomnia itself [18–20]. Another approach is shifting the research focus to presumed mechanisms underlying subjective measures. How do patients with insomnia evaluate their sleep? Why are there differences between patients and HGS regarding processing of equivalent sleep-related stimuli, and even generally aversive stimuli [21]?

## Objective of the Review

To address these questions and to gain a deeper understanding of the disorder in general, the potential impairment of affect and hyperarousal in patients with insomnia [22, 23] should be taken into account. Considering subjective measures as a result of a complex cognitive sequence of perception, processing, appraisal and retrieval, they might be highly biased by affect and arousal. Following this line of thought, objective counterparts to subjective impairments in insomnia are most likely to be found by examining affect and arousal differences between patients and HGS. Since these features also appear to be associated with the predictive value of insomnia for depression [6, 7], a review on their neurobiological correlates seems important—even though a general meta-analysis (no focus on structures related to affect and arousal) by Tahmasian et al. [24] suggests that there are no consistent brain alterations in insomnia. Specifically, we reviewed neuroimaging studies of the amygdala in relation to the proposed Hyperarousal Theory of insomnia as well as neuroimaging studies that investigated changes in the default mode network and salience network in relation to arousal and emotional memory.

## Neuroimaging Studies on the Amygdala

### The Hyperarousal Theory

A prominent model of insomnia including affect and arousal as key elements is the *Hyperarousal Theory* (HT), comprehensively described in Riemann et al. [22]. On a cognitive-behavioural level, insomnia is assumed to emerge from dysfunctional learning processes: Hereby, overly intensive cognitive processing of the ongoing, past or future sleep disturbance leads to strong aversive reactions towards sleep and sleep-related stimuli. In turn, sleep disturbances increase, which then again strengthens aversive reactions, and so on. Eventually, fear conditioning consolidates, generalizes and, in consequence, is accompanied by feelings of helplessness, extensive worrying, dysfunctional coping strategies (e.g. withdrawal or substance abuse) and a permanent state of increased arousal [22]. On endocrine and physiological levels, increased arousal in patients with insomnia has been associated with increased cortisol secretion [25], alterations in nocturnal EEG beta power [26] and heart rate variability [27]. Also, processes described in the HT are partly supported by neuroimaging studies; however, there are inconsistencies contradicting the assumption that patients with insomnia are in a permanent, global state of hyperarousal (for a review with a focus on functional neuroimaging, see [23•]). Yet, various neuroimaging studies on brain areas associated with affective cognition suggest impairments in patients with insomnia (e.g. atrophic hippocampal substructures, see [28]; dysfunctional prefrontal activity, see [29]).

### Amygdala Reactivity, Morphometry and Adaptation

The amygdala plays a crucial role in processing and memorizing arousing and fear-inducing stimuli [30, 31]. Moreover, it is discussed as an important link between insomnia and depression [8•]. Given these facts, the region might be of particular interest in insomnia from a HT perspective. So far, the only neuroimaging study focussing explicitly on amygdala reactivity (AR) in patients with insomnia vs. HGS was conducted by Baglioni et al. [32]. The authors presented sleep-related and non-sleep-related pictures of different valence (neutral or negative) and arousal level, while measuring amygdala reactivity by means of functional magnetic resonance imaging (fMRI). Surprisingly, only HGS showed increased AR for negative non-sleep-related stimuli (compared with neutral non-sleep-related stimuli). However, a reverse effect was found for negative sleep-related stimuli: AR turned out to be stronger in patients with insomnia compared with HGS. Although no interaction effect was found, the results might be interpreted as dysfunctional narrowing of affective cognition: Sleep-related negative stimuli produce an overly strong reaction while negative non-sleep-related stimuli are neglected. Since patients with insomnia are confronted with aversive, sleep-related questions during data acquisition, this could be one explanation for the previously mentioned discrepancy between objective and subjective measures of sleep disturbances. It must be remarked, however, that Spiegelhalder et al. [33], who presented sleep-related words (e.g. “tired”, “bed”, “night”) instead of pictorial stimuli in their experimental design, did not find any between-group AR differences in the same sample of patients with insomnia as Baglioni et al. [32]. Possibly, this might be explained with reduced stimuli intensity compared with Baglioni et al. [32]; nonetheless, future research on AR in insomnia is needed.

Following a morphometric perspective, a recent study by Gong et al. [34] suggests no general amygdala volume alteration in patients with insomnia. However, shape analyses showed localized amygdala atrophies (left: superficial and basolateral nuclei; right: basolateral nuclei). These results might be in line with reactivity findings suggesting that patients with insomnia do not show an amygdala hypofunction per se but rather a specific dysfunctional respectively atrophic pattern. Aptly, the association between altered amygdala morphology and psychological constructs relevant for affective cognition (frontal function, memory) is supported by Koo et al. [35•] who observed an association between subcortical atrophic shape changes and cognitive decline in patients with insomnia.

A recent study by Wassing et al. [36••] examined amygdala function with respect to alterations in REM sleep. In their experiment, subjects were presented with shame-inducing stimuli (unrelated to sleep) during an fMRI scan before going to bed and after waking up. Their results suggest reduced amygdala adaptation (= degree of amygdala reactivity decrease overnight) in persons with restless REM sleep—a phenomenon that appears to be tightly associated with insomnia disorder [37] (for an overview, see [13]), and with impairment of affective processes in general [38]. Since the study design does not allow a comparison between patients with insomnia and HGS (no grouping), it is problematic to derive insomnia-specific implications. However, the results by Wassing et al. [36••] might implicate an association between REM sleep disturbances—a presumable feature of insomnia—and impaired sleep-related brain plasticity.

Summarizing, there are indications that the amygdala plays an important role in impaired affective cognition in patients with insomnia: There might be (1) an increased reactivity to negative sleep-related stimuli, (2) a decreased reactivity to non-sleep-related stimuli and (3) localized atrophies but no overall volume reduction on a morphometric level. Furthermore, there might be (4) a general difficulty in adapting to negative stimuli after sleep disturbances. It could be hypothesized that the latter is particularly relevant for sleep-related stimuli in patients with insomnia, given the assumption that reactivity to non-sleep-related stimuli (next to sleep-related stimuli) is minimal in the first place (see point 1).

## Resting-State Functional MRI in Insomnia

### The Default Mode Network

Another main approach towards examining affect and arousal in insomnia by means of neuroimaging is resting-state fMRI (RS-fMRI, for a systematic review, see [39••]). The most common method for analyzing RS-fMRI data is functional connectivity (FC) analysis, focussing on the relationship between BOLD signals obtained from different brain regions. At that, the most frequently applied procedure is seed-based correlation analysis, which displays the whole brain connectivity pattern of a region of interest or seed region, revealing the network of regions most strongly functionally connected to it [40]. To assess resting state connectivity, subjects are instructed to lie still, think of nothing in particular and try not to fall asleep—the latter is ideally monitored via EEG [41]. RS-fMRI findings give insight about the inherent organization and functioning of the brain and about how communication between different regions might be altered in a particular disorder. A prominent connectivity pattern during resting state is the default mode network (DMN), involving inter alia the anterior and posterior cingulate cortex, the inferior parietal cortex, the ventromedial prefrontal cortex, the retrosplenial cortex, the precuneus and the hippocampus [42, 43]. The DMN is assumed to be associated with self-referential processing and emotion regulation [44] and was found to play an important role in dysfunctional affective cognition in depression [45].

Regen et al. [46•] examined how altered DMN connectivity during resting state is associated with insomnia disorder. Comparing patients with insomnia and HGS by means of RS-fMRI and FC analysis (posterior cingulate cortex as seed region), no significant differences in DMN connectivity between both groups were reported. However, a positive association between polysomnographically determined sleep continuity and sleep architecture disturbances and DMN connectivity (especially between the retrosplenial cortex and hippocampus) was found in an exploratory approach conducting FC analysis with hippocampus as seed region. These results might indicate that waking resting-state connectivity between hippocampus and other DMN regions is related to sleep disturbances in insomnia disorder. Admittedly, this interpretation has to be treated with caution since it is based upon post hoc analyses only.

In a more recent RS-fMRI study by Leerssen et al. [47••], connectivity between hippocampus and other brain regions in patients with insomnia vs. HGS was examined with a relatively large sample size (*n* = 65 per group). Conducting FC analysis with hippocampus as seed region, increased connectivity between hippocampus (bilateral) and a cluster of voxels in the left middle frontal gyrus (MFG) was found. In addition, increased strength of hippocampus-MFG connectivity was related to increased subjective insomnia severity, to lower subjective sleep efficiency, to a shorter total sleep time and to an increase in wake time after sleep onset. Interpreting these outcomes with respect to the DMN concept, the results might implicate disturbed self-related processing and emotion regulation deficits in patients with insomnia. Notably, rumination, which is considered a maladaptive emotion regulation strategy [48], appears to be associated with increased DMN connectivity in general and hippocampus-MFG connectivity in particular [49, 50]. This type of dysfunctional affective cognition is assumed to play a central role in maintaining processes underlying chronic insomnia disorder [51] and is an important element in HT [1, 22]. Furthermore, it must be complemented that hippocampal regions seem to be coupled with other DMN structures particularly during episodic memory retrieval but not during encoding [52]. Considering the tight interplay between hippocampus and amygdala [53], the DMN findings by Leerssen et al. [47••] could be interpreted in line with the amygdala adaptation findings by Wassing et al. [36••] and with the fear conditioning hypothesis included in the HT: Patients with insomnia might be prone to dysfunctional learning processes since extinction of conditioned fear reaction is obstructed by enhanced recall of negative experiences and maladaptive emotion regulation.

### The Limbic Circuit

A recent fMRI study examining these aspects of insomnia was conducted by Wassing et al. [54••]: Patients with insomnia and HGS were compared with respect to their responses to novel vs. relived negative experiences. On an operational level, a novel negative experience was created by confronting subjects with a shame-inducing recording of themselves singing karaoke, whereas a relived negative experience was created by instructing subjects to bring previously specified, troublesome biographic events to their minds. In line with the conclusions drawn above, both groups differ exclusively in responses to relived experiences: fMRI results suggest that patients with insomnia experience reliving emotional distress from the distant past more intensively due to strong involvement of the limbic circuit, in particular of the anterior cingulate cortex (ACC). The limbic circuit is assumed to play a crucial role in encoding and retrieval of novel emotional memory; however, it is assumed to become less involved over time through consolidation and neocortical integration processes [55–57]. This does not seem to be the case in patients with insomnia: The ACC remains involved when reliving emotional experiences, even if these lie in the distant past.

As multiple studies suggest [32, 34–36, 46•, 47•], functioning and physiology of amygdala and hippocampus might be altered in patients with insomnia. Finding differences in ACC dissociation from long-term memory traces between HGS and patients with insomnia, Wassing et al. [54••] add another primary limbic structure to the list. Interestingly, the ACC has previously been found to be bigger [58] and to display a reduced level of inhibitory γ-aminobutyric acid [59] in patients with insomnia. Although morphometric and spectroscopic MRI studies on insomnia remain inconsistent to this day, the role of the ACC seems worth being examined in further research.

### The Salience Network

While the hippocampus is regarded as part of the DMN, the ACC and the amygdala are considered to comprise key nodes of the salience network (SN), which is assumed to be involved in the, detection of behaviourally relevant stimuli and the coordination of neural resources “[39••]. The latter function particularly includes switching between the DMN and the central executive network in response to salient stimuli [60]. Besides processing of external (e.g. amygdala reactivity towards sleep-related stimuli) and internal stimuli (e.g. ACC involvement during reliving negative experiences), functionality of the SN is assumed to also cover integration of emotional and physiological states [60, 61]. At this, the insular cortex (IC) and its connectivity within the SN might play a particularly important role. Comparing HGS and patients with insomnia during resting state, Chen et al. [61] found that IC connectivity within the SN is increased in patients with insomnia and, furthermore, is associated with negative affect. It might be concluded that altered insula activation contributes to subjective distress and a poor sleep experience in insomnia. The tight relation between impaired IC functioning and disordered sleep is also suggested by Koenigs et al. [62]: In their study, they found an association between insomnia symptoms and left IC lesions in war veterans.

**Fig. 1:**
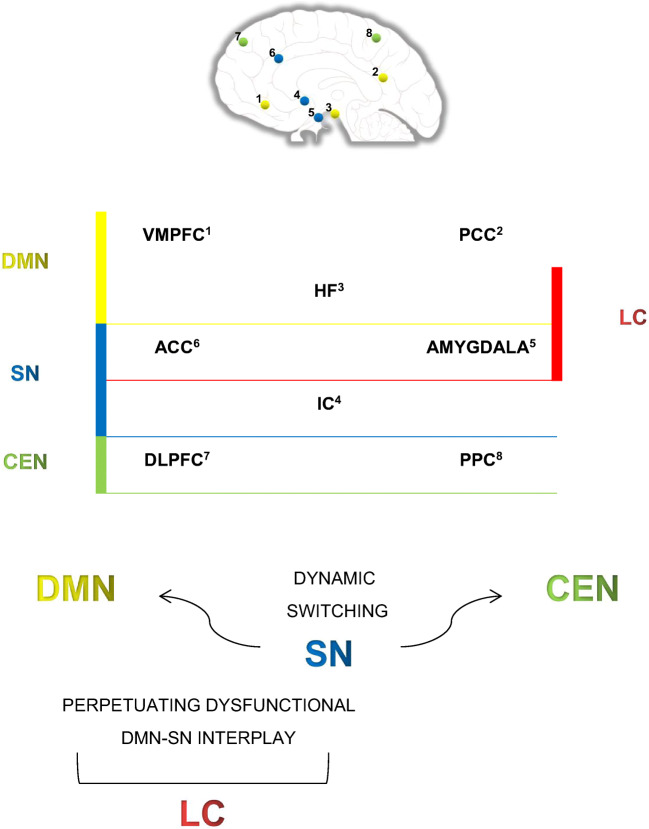
Illustration of linkages between primary limbic structures and the default mode network, respectively, the salience network as described in the summarizing paragraph of the current section. Abbreviations: DMN, default mode network; SN, salience network; CEN, central executive network; LC, limbic circuit; VMPFC, ventromedial prefrontal cortex; PCC, posterior parietal cortex; HF, hippocampal formation; ACC, anterior cingulate cortex; IC, insular cortex; DLPFC, dorsolateral prefrontal cortex; PCC, posterior cingulate cortex

Summarizing, it can be assumed that the DMN, the SN and also the limbic circuit—comprising key nodes of both networks—are neural systems of particular interest for research on affect and arousal in insomnia disorder (see Fig. 1). Increased DMN connectivity in insomnia seems to be associated with subjective sleep disturbances, dysfunctional coping with hyperarousal, maladaptive emotion regulation (particularly rumination) and enhanced (negative) memory retrieval. Increased SN connectivity in insomnia is suggested to be associated with overly sensitive detection of negative internal and external stimuli, disturbed integration of emotional and physiological states as well as ill-coordination of neural resources. Although the role of the limbic circuit as a link between the DMN and the SN is not fully understood yet, it might be assumed that primary limbic structures are key elements in perpetuating the impaired interplay between both networks. As has been suggested, dysfunctional learning processes in patients with insomnia involve increased amygdala reactivity, decreased amygdala adaptation and adverse modulation of hippocampal-dependent memories. Moreover, obstructed extinction of conditioned fear reactions in insomnia seems to be associated with enhanced recall of negative experiences (persisting ACC activation). Apparently, the limbic circuit is involved in all of these maintaining processes considered in insomnia.

## Conclusions

Recent neuroimaging studies examined in how far insomnia should be considered as a disorder of affect and arousal, characterized by biased appraisal of internal and external stimuli as well as dysfunctional learning behaviour. Playing a crucial role in processing and memorizing arousing and fear-inducing stimuli, the amygdala has been subject of such examinations: It was found that patients with insomnia show increased amygdala reactivity towards negative sleep-related stimuli [32], amygdala shape alterations [34, 35] and a reduced overnight amygdala adaptation following restless REM sleep [36••]. Conclusively, these findings suggest that processing of and learning from negative sleep-related stimuli might be intensified in patients with insomnia regarding quality and persistence of affect and arousal. A further approach comprises recent studies on RS-fMRI: Regen et al. [46•] found a positive relation between sleep disturbances and hippocampal connectivity within the DMN, a neural network associated with self-related processing and emotion regulation. Complementarily, Leerssen et al. [47••] observed increased hippocampus-MFG connectivity in patients with insomnia—a pattern associated with rumination and episodic memory retrieval. With respect to the latter aspect, Wassing et al. [54••] describe that ACC activation remains involved when patients with insomnia relive emotional distress from the distant past. Since ACC and the amygdala are assumed to comprise key nodes of it, the SN might play an important role in insomnia. Findings that IC connectivity within the SN seems to be increased in patients with insomnia [61] strengthen this assumption.

Taken together, it can be assumed that dysfunctional detection, processing, appraisal and memorizing of negative internal and external (sleep-related) stimuli as well as maladaptive emotion regulation strategies are key components of insomnia and tightly connected to subjective impairment of sleep and daytime functioning. Although neuroimaging studies still remain inconsistent in some respects, there is growing evidence that primary limbic structures—in particular by being overly connected within the DMN, respectively, within the SN—might not only contribute to dysregulated affect and arousal in insomnia but also play a crucial role in maintaining processes like dysfunctional learning and enhanced recall of negative experiences. Future research should more and more carve out the exact role of these systems, while also examining the interplay between them. For example, it would be instructive to explore in how far switching between DMN and SN is aberrant in patients with insomnia compared with HGS.

From a clinical perspective, recent findings strengthen the idea of integrating emotion regulation training into psychotherapeutic treatment of insomnia [63]. However, a positive effect of Cognitive Behavioural Therapy of Insomnia (CBT-I) on altered neurobiological processes has so far been suggested only by few studies (reduced activation in response to sleep-related stimuli, see [64]; reduced FC of subcortical with cortical regions during resting state, see [65]). It might therefore be helpful to further examine the association between insomnia symptoms and neurobiological correlates over time e.g. in longitudinal intervention studies. Given the assumption that subjective impairment is closely linked to dysfunctional affective cognition, patients with insomnia might benefit considerably from therapeutic interventions addressing affect regulation.
